# Supplementary material for: Biomedical Evaluation of Early Chronic Kidney Disease in the Air Force: Building a Predictive Model from the Taiwan Military Health Service
Source: Bioengineering (Basel). 2024 Feb 28;11(3):231. doi: 10.3390/bioengineering11030231 (PMC10968100; doi:10.3390/bioengineering11030231)
Supplement: Supplementary file 1 [file bioengineering-11-00231-s001.zip › bioengineering-2825752-supplementary.pdf]

Supplementary Table S1. Demographic characteristics (N=212).

Item		Number of people	Percentage (%)	Mean ± SD
Age group				40.4±7.15
	30-39 years	100	47.2	
	40-49 years	82	38.7	
	over 50 years	30	14.2	
Hospital performing physical examination				
	Taoyuan Armed Forces General Hospital (Hsinchu Branch)	50	23.6	
	Taichung Armed Forces General Hospital	23	10.8	
	Kaohsiung Armed Forces General Hospital (Gangshan Branch)	126	59.4	
	Hualien Armed Forces General Hospital	13	6.1	
Unit location				
	North	50	23.6	
	Middle	23	10.8	
	South	126	59.4	
	Huadong	13	6.1	
Aircraft type				
	Fighter [a]	121	57.1	
	Non-fighter [b]	91	42.9	

a. Fighters: Mirage 2000, F16, AIDC F-CK-1 Ching-kuo, F5E/F
b. Non-fighters: Aircrafts other than fighters and other units

Supplementary Table S2. The results of urinalysis (N=212).

Item		Number of people	Percentage	Abnormal indicators
Urine protein	Normal	197	92.9	
	Abnormal	15	7.1	Positive
Urine sugar	Normal	212	100.0	
	Abnormal	0	0	Positive
Urobilinogen	Normal	194	91.5	
	Abnormal	18	8.5	Positive
Bilirubin in urine	Normal	201	94.8	
	Abnormal	11	5.2	Positive
Ketones in urine	Normal	194	91.5	
	Abnormal	18	8.5	Positive
Urine pH	Normal	211	99.5	
	Abnormal	1	0.5	>8.0, <5.0
Urine specific gravity	Normal	208	98.1	
	Abnormal	4	1.9	>1.030, <1.005
Urine occult blood	Normal	186	87.7	
	Abnormal	26	12.3	Positive
Urine WBCs	Normal	201	94.8	
	Abnormal	11	5.2	Positive
Urine RBCs	Normal	210	99.1	
	Abnormal	2	0.9	Positive

Supplementary Table S3. Analysis of complete blood count tests (N=212).

Item (unit)		Number of people [a] (%)	Mean ± SD	Abnormal indicators
WBC count (10^3/μl)			6.66±1.62	
	Normal	203(95.8)		
	Abnormal	9(4.2)		> 11.00, <4.50
RBC count (10^6/μl)			5.19±0.45	
	Normal	187(88.2)		
	Abnormal	25(11.8)		> 6.00, <4.60
Haemoglobin (g/dl)			15.66±0.96	
	Normal	205(96.7)		
	Abnormal	7(3.3)		> 18.0, <13.5
Hct (%)			45.32±2.48	
	Normal	205(96.7)		
	Abnormal	7(3.3)		> 54.0, <40.0
MCH (pg)			30.39±2.59	
	Normal	153(93.9)		
	Abnormal	10(6.1)		> 34.6, <25.4
MCV (fl)			87.69±6.68	
	Normal	198(93.4)		
	Abnormal	14(6.6)		> 100.0, <79.0
MCHC (g/dl)			34.50±0.93	
	Normal	160(98.8)		
	Abnormal	2(1.2)		> 37.0, <30.0
PLT (10^3/μl)			237.44±51.30	
	Normal	205(96.7)		
	Abnormal	7(3.3)		> 400, <150

a. If the number of people for one item is not equal to the total number, people are missing for this item, and thus, the effective percentage is presented.

Supplementary Table S4. Analysis of blood biochemical tests of Air Force aircrew (N=212).

Item (unit)		Number of people (%)	Mean ± SD	Abnormal indicators
FBG (mg/dl)			97.08±9.67	
	Normal	138(65.1)		
	Abnormal	74(34.9)		≥100
BUN (mg/dl)			13.73±2.95	
	Normal	208(98.1)		
	Abnormal	4(1.9)		>20, <6
SCr (mg/dl)			0.98±0.14	
	Normal	191(90.1)		
	Abnormal	21(9.9)		≥1.2
Uric acid (mg/dl)			6.10±1.18	
	Normal	169(79.7)		
	Abnormal	43(20.3)		≥7.0
AST/SGOT (U/l)			25.18±10.07	
	Normal	199(94.3)		
	Abnormal	12(5.7)		≥40
ALT/SGPT (U/l)			32.94±25.60	
	Normal	168(79.6)		
	Abnormal	43(20.4)		≥41
ALP (U/l)			65.11±16.66	
	Normal	212(100.0)		
	Abnormal	0(0)		≥130
TP (g/dl)			7.38±0.40	
	Normal	209(98.6)		
	Abnormal	3(1.4)		>8.7, <6.6
Alb (g/dl)			4.70±0.25	
	Normal	183(86.3)		
	Abnormal	29(13.7)		>4.94, <3.97
TBIL (mg/dl)			0.76±0.37	
	Normal	185(87.3)		
	Abnormal	27(12.7)		≥1.2
TG (mg/dl)			122.50±66.54	
	Normal	155(73.1)		
	Abnormal	57(26.9)		≥150
TCH (mg/dl)			191.29±27.57	
	Normal	136(64.2)		
	Abnormal	76(35.8)		≥200
HDL-C (mg/dl)			50.27±13.00	
	Normal	164(77.4)		
	Abnormal	48(22.6)		<40 (male)
LDL-C (mg/dl)			125.62±26.27	
	Normal	122(57.5)		
	Abnormal	90(42.5)		≥130

Supplementary Table S5a. Comparison of different serum creatinine (SCr) concentrations and basic examinations (N=212).

Item	Group	Quartile 1 (n=47) Number of people [a] (%)	Quartile 1 (n=47) mean ±SD	Quartile 2 (n=55) Number of people [a] (%)	Quartile 2 (n=55) mean ± SD	Quartile 3 (n=55) Number of people [a] (%)	Quartile 3 (n=55) mean ± SD	Quartile 4 (n=55) Number of people [a] (%)	Quartile 4 (n=55) mean ± SD	*p* value	*p* for trend
Age			40.60±6.95		38.76±5.74		40.85±7.89		41.56±7.70	0.205	0.225
	30-39 years	22(46.8)		30(54.5)		27(49.1)		21(38.2)			
	40-49 years	18(38.3)		22(40.0)		17(30.9)		25(45.5)		0.266	
	Over 50 years	7(14.9)		3(5.5)		11(20.0)		9(16.4)			
BMI			25.15±2.22		25.03±2.40		25.33±1.05		24.86±2.25	0.976	0.811
SBP			126.81±6.15		126.04±8.37		131.00±6.54		129.54±8.30		0.207
	Normal	9(56.3)		13(59.1)		1(16.7)		5(45.5)		0.294	
	Abnormal	7(43.8)		9(40.9)		5(83.3)		6(64.5)			
DBP			79.50±5.44		79.04±6.60		80.50±7.53		80.72±6.97	0.894	0.543
	Normal	12(75.0)		17(77.3)		3(50.0)		8(72.7)		0.608	
	Abnormal	4(25.0)		5(22.7)		3(50.0)		3(27.3)			
BP	Normal	9(56.3)		12(54.5)		1(16.7)		5(45.5)		0.366	
	Hypertension	7(43.8)		10(45.5)		5(83.3)		6(54.5)			
aMDRD (ml/min/1.73 m^2)											
	Non-CKD	43(91.5)		47(85.5)		52(94.5)		50(90.9)		0.431	
	CKD	4(8.5)		8(14.5)		3(5.5)		5(9.1)			

a. If the number of people for one item is not equal to the total number, people are missing for this item, and thus, the effective percentage is presented
Continuous variable: the *p* value was obtained by analysis of variance (ANOVA) and linear regression; Categorical variable: the *p* value is obtained by the two-tailed chi-square test

Supplementary Table S5b. Comparison of different serum creatinine (SCr) concentrations and urinalysis (N=212).

Item	Group	Quartile 1 (n=47)		Quartile 2 (n=55)		Quartile 3 (n=55)		Quartile 4 (n=55)		*p* value	*p* for trend
		Number of people [a] (%)	mean ± SD	Number of people [a] (%)	mean ± SD	Number of people [a] (%)	mean ± SD	Number of people [a] (%)	mean ± SD		
Urine protein	Normal	43(91.5)		49(89.1)		52(94.5)		53(96.4)		0.460	
	Abnormal	4(8.5)		6(10.9)		3(5.5)		2(3.6)			
Urobilinogen	Normal	46(97.9)		47(85.5)		50(90.9)		51(92.7)		0.159	
	Abnormal	1(2.1)		8(14.5)		5(9.1)		4(7.3)			
Bilirubin in urine	Normal	47(100.0)		53(96.4)		52(94.5)		49(89.1)		0.089	
	Abnormal	0(0)		2(3.6)		3(5.5)		6(10.9)			
Ketones in urine	Normal	46(97.9)		53(96.4)		48(87.3)		47(85.5)		0.046	
	Abnormal	1(2.1)		2(3.6)		7(12.7)		8(14.5)			
Urine pH			5.93±0.77		6.09±0.80		5.71±0.66		5.72±0.77	0.026	0.029
	Normal	47(100.0)		54(98.2)		55(100.0)		55(100.0)		0.412	
	Abnormal	0(0)		1(1.8)		0(0)		0(0)			
Urine WBCs	Normal	46(97.9)		51(92.7)		53(96.4)		51(92.7)		0.545	
	Abnormal	1(2.1)		4(7.3)		2(3.6)		4(7.3)			
Urine occult blood	Normal	37(78.7)		43(78.2)		53(96.4)		53(96.4)		0.001	
	Abnormal	10(21.3)		12(21.8)		2(3.6)		2(3.6)			
Urine RBCs	Normal	46(97.9)		54(98.2)		55(100.0)		55(100.0)		0.531	
	Abnormal	1(2.1)		1(1.8)		0(0)		0(0)			

a. If the number of people for one item is not equal to the total number, people are missing for this item, and thus, the effective percentage is presented
Continuous variable: the *p* value is obtained by analysis of variance (ANOVA) and linear regression; Categorical variable: the *p* value is obtained by the two-tailed chi-square test

Supplementary Table S5c. Comparison of different serum creatinine (SCr) concentrations and blood biochemical tests (N=212).

Item	Group	Quartile 1 (n=47)		Quartile 2 (n=55)		Quartile 3 (n=55)		Quartile 4 (n=55)		*p* -value	*p* for trend
		Number of people[a] (%)	mean±SD	Number of people[a] (%)	mean±SD	Number of people[a] (%)	mean±SD	Number of people[a] (%)	mean±SD		
FBG			93.55±12.01		97.77±9.46		99.53±8.60		96.94±7.84	0.017	0.065
	Normal	33(70.2)		38(69.1)		29(52.7)		38(69.1)		0.171	
	Abnormal	14(29.8)		17(30.9)		26(47.3)		17(30.9)			
BUN			12.92±2.38		13.05±3.38		14.15±2.77		14.68±2.84	0.004	<0.001
	Normal	47(100.0)		54(98.2)		54(98.2)		53(96.4)		0.611	
	Abnormal	0(0)		1(1.8)		1(1.8)		2(3.6)			
Uric acid			5.90±1.16		5.92±1.15		5.87±1.02		6.69±1.20	<0.001	0.001
	Normal	40(85.1)		45(81.8)		47(85.5)		37(67.3)		0.061	
	Abnormal	7(14.9)		10(18.2)		8(14.5)		18(32.7)			
ALT (SGPT)			39.78±37.60		32.90±24.24		32.19±19.25		27.89±18.17	0.136	0.025
	Normal	35(74.5)		42(77.8)		43(78.2)		48(87.3)		0.401	
	Abnormal	12(25.5)		12(22.2)		12(21.8)		7(12.7)			
TBIL			0.71±0.26		0.77±0.40		0.82±0.45		0.73±0.35	0.553	0.454
	Normal	43(91.5)		49(89.1)		46(83.6)		47(85.5)		0.630	
	Abnormal	4(8.5)		6(10.9)		9(16.4)		8(14.5)			
TG			122.42±59.10		129.01±67.12		110.73±46.13		127.84±86.47	0.460	0.957
	Normal	34(72.3)		38(69.1)		42(76.4)		41(74.5)		0.845	
	Abnormal	13(27.7)		17(30.9)		13(23.6)		14(25.5)			
TCH			190.73±28.90		193.15±24.56		190.52±30.52		190.48±26.82	0.953	0.830
	Normal	32(68.1)		37(67.3)		33(60.0)		34(61.8)		0.779	
	Abnormal	15(31.9)		18(32.7)		22(40.0)		21(38.2)			
HDL-C			50.67±12.84		49.10±13.24		49.98±12.99		51.39±13.14	0.821	0.672
	Normal	36(76.6)		44(80.0)		40(72.7)		44(80.0)		0.770	
	Abnormal	11(23.4)		11(20.0)		15(23.7)		11(20.0)			
LDL-C			130.21±27.34		130.44±25.15		123.93±25.93		118.61±25.69	0.058	0.010
	Normal	25(53.2)		29(52.7)		31(56.4)		37(67.3)		0.384	
	Abnormal	22(46.8)		26(47.3)		24(43.6)		18(32.7)			

a. If the number of people for one item is not equal to the total number, people are missing for this item, and thus, so the effective percentage is presented

Continuous variable: the *p* value is obtained by analysis of variance (ANOVA) and linear regression; Categorical variable: the *p* value is obtained by the two-tailed chi-square test.

Supplementary Table S6a. Correlation between age and CKD (N=212).

Item	Group	30-39 years old (n=100) Number of people (%) [a] mean ± SD	40-49 years old (n=82) Number of people (%) [a] mean ± SD	>50 years old (n=30) Number of people (%) [a] mean ± SD	*p* value	*p* for trend
Estimated GFR (eGFR) (ml/min/1.73 m^2)		96.29±14.53	90.43±15.89	90.05±19.34	0.025	0.013
	≥90	73(73.0)	36(43.9)	10(33.3)		
	60-89	26(26.0)	44(53.7)	20(66.7)	<0.001	
	45-59	1(1.0)	2(2.4)	0(0)		
CKD						
	Non-CKD	88(88.0)	74(90.2)	30(100.0)	0.142	
	CKD	12(12.0)	8(9.8)	0(0)		
CKD stage						
	Stage 1	8(8.0)	2(2.4)	0(0)		
	Stage 2	3(3.0)	4(4.9)	0(0)	0.182[c]	
	Stage 3	1(1.0)	2(2.4)	0(0)		

a. If the number of people for one item is not equal to the total number, people are missing for this item, and thus, the effective percentage is presented
Continuous variable: the *p* value is obtained by analysis of variance (ANOVA) and linear regression; Categorical variable: the *p* value is obtained by the two-tailed chi-square test
c. The *p* value of CKD stage in the >50 year age group is not presented

Supplementary Table S6b. Correlation between the age and urinalysis (N=212).

Item	Group	30-39 years old (n=100)[a]		40-49 years old (n=82)[a]		>50 years old (n=30)[a]		*p* value	*p* for trend
		Number of people (%)	mean±SD	Number of people (%)	mean±SD	Number of people (%)	mean±SD		
Urine protein	Normal	90(90.0)		77(93.9)		30(100.0)		0.157	
	Abnormal	10(10.0)		5(6.1)		0(0)			
Urobilinogen	Normal	90(90.0)		75(91.5)		29(96.7)		0.517	
	Abnormal	10(10.0)		7(8.5)		1(3.3)			
Bilirubin in urine	Normal	95(95.0)		78(95.1)		28(93.3)		0.925	
	Abnormal	5(5.0)		4(4.9)		2(6.7)			
Ketones in urine	Normal	94(94.0)		73(89.0)		27(90.0)		0.463	
	Abnormal	6(6.0)		9(11.0)		3(10.0)			
Urine pH			5.93±0.69		5.86±0.86		5.61±0.69	0.134	0.061
	Normal	100(100.0)		81(98.8)		30(100.0)		0.451	
	Abnormal	0(0)		1(1.2)		0(0)			
Urine WBCs	Normal	95(95.0)		76(92.7)		30(100.0)		0.301	
	Abnormal	5(5.0)		6(7.3)		0(0)			
Urine occult blood	Normal	83(83.0)		74(90.2)		29(96.7)		0.091	
	Abnormal	17(17.0)		8(9.8)		1(3.3)			
Urine RBCs	Normal	98(98.0)		82(100.0)		30(100.0)		0.323	
	Abnormal	2(2.0)		0(0)		0(0)			

a. If the number of people for one item is not equal to the total number, people are missing for this item, and thus, the effective percentage is presented
Continuous variable: the *p* value is obtained by analysis of variance (ANOVA) and linear regression; Categorical variable: the *p* value is obtained by the two-tailed chi-square test

Supplementary Table S6c. Correlation between age and the blood biochemical tests (N=212).

Item	Group	30-39 years old (n=100) [a]		40-49 years old (n=82) [a]		>50 years old (n=30) [a]		p value	p for trend
		Number of people (%)	mean ± SD	Number of people (%)	mean ± SD	Number of people (%)	mean ± SD		
FBG			95.40±8.46		97.77±10.92		100.75±8.82	0.020	0.005
	Normal	73(73.0)		52(63.4)		13(43.3)		0.011	
	Abnormal	27(27.0)		30(36.6)		17(56.7)			
BUN			13.72±3.12		13.60±2.62		14.06±3.28	0.767	0.736
	Normal	97(97.0)		82(100.0)		29(96.7)		0.275	
	Abnormal	3(3.0)		0(0)		1(3.3)			
SCr			0.97±0.13		0.99±0.15		0.96±0.15	0.671	0.928
	Normal	92(92.0)		71(86.6)		28(93.3)		0.388	
	Abnormal	8(8.0)		11(13.4)		2(6.7)			
Uric acid			6.19±1.08		6.08±1.31		5.86±1.09	0.393	0.182
	Normal	76(76.0)		68(82.9)		25(83.3)		0.445	
	Abnormal	24(24.0)		14(17.1)		5(16.7)			
ALT (SGPT)			34.66±29.64		30.54±18.88		33.85±27.45	0.550	0.590
	Normal	77(77.8)		67(81.7)		24(80.0)		0.807	
	Abnormal	22(22.2)		15(18.3)		6(20.0)			
TBIL			0.75±0.37		0.79±0.38		0.74±0.34	0.748	0.833
	Normal	92(92.0)		67(81.7)		26(86.7)		0.116	
	Abnormal	8(8.0)		15(18.3)		4(13.3)			
TG			113.54±47.99		133.67±86.45		121.88±53.36	0.127	0.203
	Normal	76(76.0)		56(68.3)		23(76.7)		0.452	
	Abnormal	24(24.0)		26(31.7)		7(23.3)			
TCH			187.06±27.28		194.51±24.56		196.61±34.43	0.100	0.041
	Normal	74(74.0)		45(54.9)		17(56.7)		0.018	
	Abnormal	26(26.0)		37(45.1)		13(43.3)			
HDLC			50.51±11.63		50.86±14.91		47.86±11.81	0.543	0.474
	Normal	80(80.0)		61(74.4)		23(76.7)		0.664	
	Abnormal	20(20.0)		21(25.6)		7(23.3)			
LDLC			123.14±26.15		127.12±24.31		129.75±31.49	0.389	0.172
	Normal	65(65.0)		41(50.0)		16(53.3)		0.111	
	Abnormal	35(35.0)		41(50.0)		14 (46.7)			

a. If the number of people for one item is not equal to the total number, people are missing for this item, and thus, the effective percentage is presented.
Continuous variable: the p value is obtained by analysis of variance (ANOVA) and linear regression; Categorical variable: the p value is obtained by the two-tailed chi-square test.

Supplementary Table S7a. Comparison of abnormalities in previous urinalysis (N=212).

Item	Group	First physical examination Number of people [a] (%)	First physical examination mean±SD	Second physical examination Number of people [a] (%)	Second physical examination mean±SD	Third physical examination Number of people [a] (%)	Third physical examination mean±SD	Fourth physical examination Number of people [a] (%)	Fourth physical examination mean±SD	*p* value	*p* for trend
Urine protein	Normal	204(96.2)		199(93.9)		200(94.3)		197(92.9)		0.513	
	Abnormal	8(3.8)		13(6.1)		12(5.7)		15(7.1)			
Urobilinogen	Normal	203(95.8)		197(92.9)		199(93.9)		194(91.5)		0.344	
	Abnormal	9(4.2)		15(7.1)		13(6.1)		18(8.5)			
Bilirubin in urine	Normal	201(94.8)		203(95.8)		199(93.9)		201(94.8)		0.857	
	Abnormal	11(5.2)		9(4.2)		13(6.1)		11(5.2)			
Ketones in urine	Normal	202(95.3)		193(91.0)		198(93.4)		194(91.5)		0.310	
	Abnormal	10(4.7)		19(9.0)		14(6.6)		18(8.5)			
Urine pH			5.81±0.79		5.82±0.81		5.84±0.79		5.86±0.76	0.917	0.489
	Normal	212(100.0)		210(99.1)		212(100.0)		211(99.5)		0.298	
	Abnormal	0(0)		2(0.9)		0(0)		1(0.5)			
Urine WBCs	Normal	205(96.7)		202(95.3)		182(85.8)		201(94.8)		<0.001	
	Abnormal	7(3.3)		10(4.7)		30(14.2)		11(5.2)			
Urine occult blood	Normal	194(91.5)		192(90.6)		183(86.3)		186(87.7)		0.283	
	Abnormal	18(8.5)		20(9.4)		29(13.7)		26(12.3)			
Urine RBCs	Normal	212(100.0)		204(96.2)		205(96.7)		210(99.1)		0.013	
	Abnormal	0(0)		8(3.8)		7(3.3)		2(0.9)			

a. If the number of people for one item is not equal to the total number, people are missing for this item, and thus, the effective percentage is presented
Continuous variable: the *p* value is obtained by analysis of variance (ANOVA) and linear regression; Categorical variable: the *p* value is obtained by the two-tailed chi-square test

Supplementary Table S7b. Comparison of abnormalities in previous blood biochemical tests (N=212).

Item	Group	First physical examination		Second physical examination		Third physical examination		Fourth physical examination		*p* value	*p* for trend
		Number of people (%)	[a] mean±SD	Number of people (%)	[a] mean±SD	Number of people (%)	[a] mean±SD	Number of people (%)	[a] mean±SD		
FBG			89.83±9.71		95.52±12.04		94.65±11.32		97.08±9.67	<0.001	<0.001
	Normal	180(84.9)		150(70.8)		146(68.9)		138(65.1)		<0.001	
	Abnormal	32(15.1)		62(29.2)		66(31.1)		74(34.9)			
BUN			14.08±3.07		13.42±2.79		14.03±2.91		13.73±0.95	0.078	0.609
	Normal	200(94.3)		208(98.1)		205(96.7)		208(98.1)		0.088	
	Abnormal	12(5.7)		4(1.9)		7(3.3)		4(1.9)			
SCr			0.88±0.14		0.90±012		0.90±0.13		0.98±0.14	<0.001	<0.001
	Normal	201(94.8)		209(98.6)		205(96.7)		191(90.1)		<0.001	
	Abnormal	11(5.2)		3(1.4)		7(3.3)		21(9.9)			
Uric acid			6.47±1.29		6.33±1.15		6.37±1.17		6.10±1.18	0.014	0.004
	Normal	142(67.0)		158(74.5)		148(69.8)		169(79.7)		0.018	
	Abnormal	70(33.0)		54(25.5)		64(30.2)		43(20.3)			
ALT(SGPT)			29.90±15.89		29.22±16.13		29.84±15.07		32.94±25.60	0.168	0.090
	Normal	169(80.1)		174(82.5)		170(81.0)		168(79.6)		0.888	
	Abnormal	42(19.9)		37(17.5)		40(19.0)		43(20.4)			
TBIL			0.88±0.37		0.87±0.40		0.83±0.41		0.76±0.37	0.056	0.010
	Normal	65(86.7)		62(79.5)		170(84.6)		185(87.3)		0.405	
	Abnormal	10(13.3)		16(20.5)		31(15.4)		27(12.7)			

TG			108.89±64.40		114.22±56.74		121.46±66.24		120.05±56.32	0.133	0.031
	Normal	173(82.8)		164(78.8)		160(76.2)		155(73.5)		0.125	
	Abnormal	36(17.2)		44(21.2)		50(23.8)		56(26.5)			
TCH			190.31±29.42		193.98±29.63		194.08±28.30		191.29±27.57	0.425	0.731
	Normal	132(62.3)		127(59.9)		126(59.4)		136(64.2)		0.732	
	Abnormal	80(37.7)		85(40.1)		86(40.6)		76(35.8)			
HDLC			51.78±12.82		51.61±13.21		51.20±12.97		50.27±13.00	0.638	0.219
	Normal	167(81.5)		171(81.4)		175(82.5)		164(77.4)		0.546	
	Abnormal	38(18.5)		39(18.6)		37(17.5)		48(22.6)			
LDLC			134.95±24.65		131.85±28.20		129.49±26.92		125.62±26.27	0.043	0.005
	Normal	34(44.7)		38(48.7)		106(52.7)		122(57.5)		0.220	
	Abnormal	42(55.3)		40(51.3)		95(47.3)		90(42.5)			

a. If the number of people for one item is not equal to the total number, people are missing for this item, and thus, the effective percentage is presented.
Continuous variable: the *p* value is obtained by analysis of variance (ANOVA) and linear regression; Categorical variable: the *p* value is obtained by the two-tailed chi-square test.
